# A Comparison of the Checklist Scoring Systems, Global Rating Systems, and Borderline Regression Method for an Objective Structured Clinical Examination for a Small Cohort in a Saudi Medical School

**DOI:** 10.7759/cureus.39968

**Published:** 2023-06-05

**Authors:** Kossay Elabd, Husam Abdul-Kadir, Abdullah Alkhenizan, Mohammed K Alkhalifa

**Affiliations:** 1 Family Medicine, King Faisal Specialist Hospital and Research Centre, Riyadh, SAU; 2 Family Medicine and Polyclinics, King Faisal Specialist Hospital and Research Centre, Riyadh, SAU

**Keywords:** tests validity and reliability, pass-fail decisions, defensible standards, borderline regression, small cohort osces (objective structured clinical examinations)

## Abstract

Background: This study aims to compare the effectiveness of using the checklist and global rating scores to evaluate the clinical competency of medical students in Objective Structured Clinical Examinations (OSCEs). Additionally, the study assesses the appropriateness of using the borderline regression method to set standards for small-scale OSCE exams and determines if the estimated passing marks differ significantly from the university's prefixed passing score of 70%. The study also examines whether the university should utilize the borderline regression method to determine passing scores for each OSCE exam instead of a set passing score.

Methods: The study analyzed medical students' grades in 11 OSCE exams in the 2022-2023 academic year at Alfaisal University, Riyadh, Saudi Arabia. Students received family medicine clerkship rotations, and after each rotation, they took an OSCE exam consisting of three stations that family medicine consultants graded. The exam included a checklist of 30 tasks and a five-level global rank scale. The study collected all the checklist marks and global rank grades and analyzed them using IBM® Statistical Package for Social Sciences (SPSS® Statistics) software. The statistical tests used were descriptive statistics, the T-test, chi-square tests, Fisher's exact test, and Pearson correlation.

Results: The study showed that students were more likely to pass when using the global rating system than the checklist scoring system. Additionally, students had a significantly lower passing rate when using the higher cut-off passing score estimated using the borderline regression method compared to the pre-set passing score of 70% established by the university (with a p-value of 0.00).

Conclusion: Each scoring system has advantages and disadvantages, but they complement each other. Combining scoring systems can produce a more comprehensive and precise evaluation of a candidate's performance. The study also emphasizes the importance of carefully selecting and validating cut-off points in OSCE exams to ensure fairness and consistency in assessment.

## Introduction

Objective Structured Clinical Examination (OSCE) is a widely used method in medical education to assess the clinical competency of medical students, residents, and healthcare professionals. Various stations are designed to evaluate a specific scenario or task the candidate must complete. The aim is to assess performance objectively and consistently, determining whether the candidate is ready to progress to the next level or practice independently [1].

Two primary methods, checklist evaluation and global rating system evaluation, have been utilized to evaluate performance in OSCE exams [2,3]. While both have shown high effectiveness, they have also exhibited drawbacks and can be unreliable when used independently [4-6].

Various methods have been used to establish reliable standards for OSCE exams, but the criteria-referenced method is most commonly used in medical education. This method utilizes a standardized scoring system based on performance to assess students' capacity to perform specific clinical tasks according to predefined criteria, or "objectives." These criteria can be derived from diverse sources, such as best practice guidelines, expert opinions, or specific learning objectives. As a result, it quantitatively measures student performance. In contrast, the global rating scoring system is another popular method utilized to assess performance subjectively based on the examiner's judgment [4-7].

Borderline regression is a standard-setting method widely used to set standards for large-scale OSCE exams. It plots the actual mark that all candidates received for a station against the global score they were given. A best-fit line is then drawn, and the point where it intersects the "borderline" indicates the cut-off mark for the checklist at that station. While the borderline regression method has better face validity, its efficacy is questioned when applied to small-scale OSCE exams [4,8-9]. 

In our family medicine clerkship program at King Faisal Specialist Hospital in Riyadh, we always used pre-determined passing checklist scores of 70% for each OSCE station without using global rating scores. The examiners rate the students based on their expected performance and knowledge on the checklist. However, in recent years, some students were observed to have received high scores with the checklist rating that they did not deserve, while other students performed well but did not receive high scores. Qualitative assessment through the incorporation of a global rating scale was thus introduced to evaluate students' performance.

This study aims to compare the pass and failure rates between global scores and checklist scores and to assess the suitability of the borderline regression method for setting standards for small-scale OSCE exams. Additionally, the research aims to determine if the borderline regression passing marks differ significantly from the university's prefixed passing score of 70%. The study will provide recommendations for the university to consider using the borderline regression method in determining passing marks for each OSCE exam.

## Materials and methods

This study examined medical students' grades who took part in 11 OSCE exams at different stations. At Alfaisal University, final-year students received family medicine clerkship rotations every academic year, and they visited our family medicine department in groups of around 25 to 30 students once a month. After each rotation, students took an OSCE exam that consisted of three OSCE stations, which family medicine consultants graded. For this study, one OSCE station was randomly selected from each of the 11 exams that took place in the 2022-2023 academic year. Students who could not complete the exam due to illness or other reasons were excluded from the study. Examiners used two scales to score candidates: the OSCE checklist, which had 30 marks available per station. Students were graded based on their performance for each checklist point, receiving a zero if they did not perform the task, a half mark if they partially completed it, and a full mark if they completed it perfectly. In addition, they used a global rank scale consisting of five levels (fail, borderline, pass, good, and excellent) based on the examiner's professional judgment of the student's performance at the end of each station (Appendix 1).

We collected all the checklist marks and the global rank grades of all the students in the 11 OSCE stations, then analyzed them using the IBM® Statistical Package for Social Sciences (SPSS®) Statistics software. Descriptive statistics were reported as percentages for categorical variables. Comparing numerical data was conducted using the t-test, while Chi-square tests and Fisher's exact test were used to compare categorical variables. Pearson's correlation was used to determine the passing score of the checklist from the global rating PassMark. The level of statistical significance was set at p<0.05. The Research Ethics Council at King Faisal Specialist Hospital in Riyadh, Saudi Arabia, approved the research. The approval number assigned by the RAC is 2221203.

## Results

The marks of 319 students from 11 OSCE stations were examined. Of this group, 56.1% of the students who took these were females, and 43.9% were males. There were no significant gender differences in those who passed the OSCE exam in either global scoring or checklist scoring (p-values of 0.325 and 0.083, respectively).

When we used the university’s pre-set pass mark of 70% for the checklist, we found that 11% of students failed their exams. When we used the global rating score, we noticed that 7.2% of the students had failed the exam; see Table 1.

**Table 1:** The student's pass/fail rate is determined using a checklist and a global scoring system.

	Checklist scoring	Percentage	Global rating scoring	Percentage
Failure	≤50%	1.6	Fail	1.6
	51-70%	9.4	Borderline	5.6
Pass	71-80%	27.3	Pass	19.4
	81-90%	37.3	Good	46.4
	90-100%	24.4	Excellent	27

**Figure 1:**
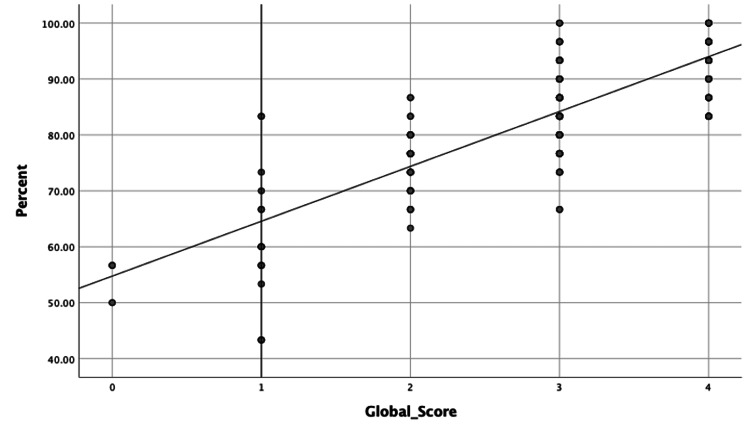
A regression analysis was conducted to determine the average scores for all of the OSCE stations and to estimate the cut-off passing score. The global rating score for each station was classified into five levels, with level one indicating failure, level two indicating borderline performance, level three indicating a passing grade, level four indicating good performance, and level five indicating excellent performance.

We utilized regression analysis to approximate the necessary passing threshold for every OSCE station. The graph illustrates that the average pass rate for all stations was 74.375% (Figure 1).

Additionally, all OSCE stations evaluated in this study displayed an estimated pass rate above the university's cut-off threshold of 70%, as seen in Table 2.

**Table 2:** Estimated passing marks for each of the 11 OSCE stations, determined through regression analysis.

Station number	Estimated pass mark
1	77.354
2	76.934
3	72.825
4	73.681
5	73.424
6	73.895
7	74.217
8	75.144
9	73.796
10	73.918
11	77.184

Fisher's exact test showed a significant difference in the distribution between passing and non-passing students when comparing the university's predetermined cut-off point of 70% with passing scores based on the borderline regression method (p-value< 0.001). If we were to use the average cut-off mark for all stations calculated using regression analysis, which is estimated to be 74.37%, 54 students (17.47%) would have failed the exam.

## Discussion

This study examined the performance scores in OSCE exams for 319 medical students who had completed their family medicine clerkship in the 2022-2023 academic year. Global rating scores and checklist scores were used to grade their performance qualitatively and quantitatively, respectively. Overall, most students performed well, and there was no significant difference in their performance scores based on gender in either of the scoring systems. However, the failure rate of students varied significantly depending on the assessment method used. When we used the global rating scoring system, the failure rate was lower at 7.2%. When we used the regression analysis method to calculate the cut-off pass mark, distinct passing scores were produced for each station, varying from 72.825% to 77.354%. All of these marks exceeded the pre-set cut-off pass mark of 70%. If we utilized the average pass mark of all stations (74.375%), calculated through the regression method, a significantly higher failure rate of 16.68% would have ensued (p-value <0.001). Establishing a defensible standard for each OSCE station is crucial to ensuring that the exam results are fair, valid, and reliable [8].

The variations we observed in pass-failure rates while using different cut-off marks, including the university's current 70% mark for the checklist, the global rating score, and the estimated pass score from regression analysis, can be attributed to various reasons [3]. Firstly, the varied difficulty levels of each station may result in varying passing grades with different scoring methods. Secondly, while the global scoring system can provide valuable insights into a student's performance, it is also subjective. Examiners might have different professional opinions about the marks the student's global performance deserves. This can result in variable global rating scores. On the other hand, although the checklist rating system is more objective, the examiner's professional opinion might be completely different about the student's performance. 

Therefore, combining both scoring methods would provide a more thorough and accurate evaluation of a candidate's performance [2,3,5-7]. In addition, to ensure fairness, the passing score for every station in the OSCE should be assessed using the borderline regression technique. This technique will help us estimate a fair cut-off passing score for every OSCE station based on its difficulty level.

Additionally, using two examiners to evaluate each station could ensure more accurate assessments. This would involve one examiner evaluating the student's performance based on the checklist while the other assesses overall performance using a global scoring system [9]. The examiners should conduct this evaluation without consulting the student's checklist score to prevent the second examiner from being influenced by the checklist mark when assigning a global rating score.

This study had limitations, including the possibility that the examiner's final assessment of a student's performance could be influenced by their score on the checklist. Additionally, although each examiner gets introduced to the learning objectives of the family medicine clerkship and to the medical school curriculum, some examiners may require further education to fully understand the expectations for students who take these exams, so the examiner's expectations should be matched to the student level. It is vital to consider a student's level of training when assessing their performance. Hence, they are not unfairly penalized or rewarded for knowledge or skills beyond their training level [9]. To ensure fairness, validity, and reliability, it is recommended that both the checklist and global rating methods be used. In addition, the borderline regression technique should be employed to determine a fair passing score for each station. Furthermore, having two independent examiners evaluate each station without being influenced by one another's scores can lead to more accurate evaluations. It will be better to recompare our checklist and global rating scores assigned to our students and redetermine their passing scores after educating our examiners about our students' expected knowledge and skills and using two examiners for each station whenever possible.

## Conclusions

The study analyzed the performance scores of medical students in OSCE exams and found that the failure rate varied significantly depending on the assessment method used. Combining the checklist and global rating scoring methods and utilizing the borderline regression technique to establish passing scores for each station could provide a more accurate and fair evaluation of a candidate's performance. Using two examiners to evaluate each station without consulting the checklist score and assessing students based on their level of training can also help ensure fair and accurate evaluations.

## Figures and Tables

**Figure 2:**
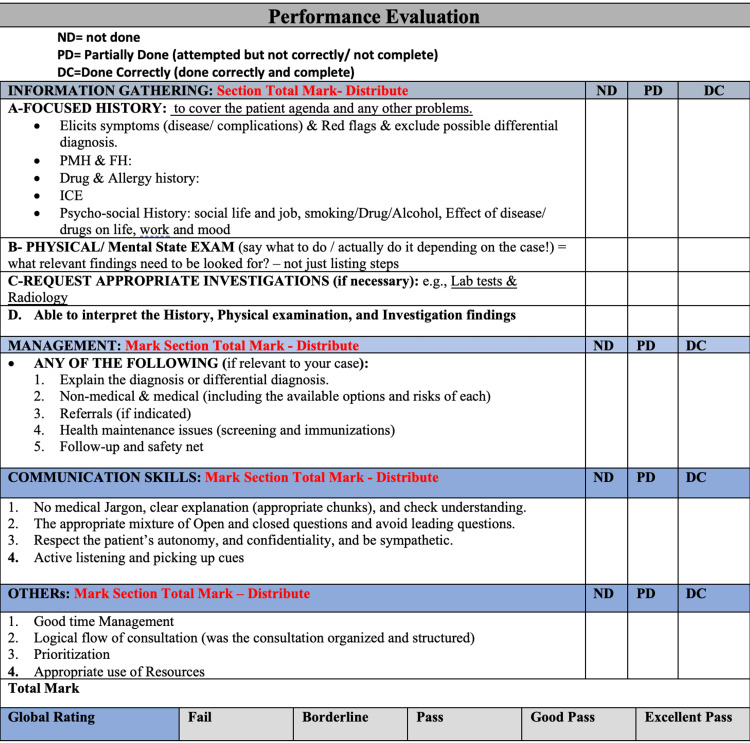
OSCE exam checklist and global rating sheet

## Appendices

Appendix 1
